# Tyraminergic and Octopaminergic Modulation of Defensive Behavior in Termite Soldier

**DOI:** 10.1371/journal.pone.0154230

**Published:** 2016-05-19

**Authors:** Yuki Ishikawa, Hitoshi Aonuma, Ken Sasaki, Toru Miura

**Affiliations:** 1 Graduate School of Environmental Science, Hokkaido University, Sapporo, Hokkaido, Japan; 2 Graduate School of Science, Nagoya University, Nagoya, Aichi, Japan; 3 Research Institute for Electronic Science, Hokkaido University, Sapporo, Hokkaido, Japan; 4 Japan Science and Technology Agency CREST, Japan Science and Technology Agency, Kawaguchi, Saitama, Japan; 5 Graduate School of Agriculture, Tamagawa University, Machida, Tokyo, Japan; University of Cologne, GERMANY

## Abstract

In termites, i.e. a major group of eusocial insects, the soldier caste exhibits specific morphological characteristics and extremely high aggression against predators. Although the genomic background is identical to the other non-aggressive castes, they acquire the soldier-specific behavioral character during the course of caste differentiation. The high aggressiveness and defensive behavior is essential for colony survival, but the neurophysiological bases are completely unknown. In the present study, using the damp-wood termite *Hodotermopsis sjostedti*, we focused on two biogenic amines, octopamine (OA) and tyramine (TA), as candidate neuromodulators for the defensive behavior in soldiers. High-performance liquid chromatographic analysis revealed that TA levels in the brain and suboesophageal ganglion (SOG) and the OA level in brain were increased in soldiers than in pseudergates (worker caste). Immunohistochemical analysis revealed that TA/OA neurons that innervate specific areas, including the mandibular muscles, antennal nerve, central complex, suboesophageal ganglion, and thoracic and/or abdominal ganglia, were enlarged in a soldier-specific manner. Together with the results that pharmacological application of TA promoted the defensive behavior in pseudergates, these findings suggest that the increased TA/OA levels induce the higher aggressiveness and defensive behavior in termite soldiers. The projection targets of these soldier-specific enlarged TA/OA neurons may have important roles in the higher aggressiveness and defensive behavior of the termite soldiers, inducing the neuronal transition that accompanies external morphological changes.

## Introduction

Division of labor has been independently acquired in various social-insect lineages, but the neurophysiological mechanisms have been studied in only a few lineages—mostly in social hymenopterans. In social animals, aggression differences among colony members are important in the division of labor [1–5]. In some social insects, including bees, aphids, and termites, individuals with unique morphologies and high aggression, i.e., soldier castes or guards, specialize in colony defense [1–3]. In almost all cases, these castes do not reproduce, and exhibit distinctive defensive behaviors against predators. The different castes of social insects share similar (sometimes identical) genetic backgrounds with other colony members, but epigenetically acquire stable high aggression and defensive behaviors during postembryonic development in a caste-specific manner [1, 2]. In termites, soldiers with constant high aggression are differentiated from workers [6–8]. Although high aggressiveness in soldiers is essential for the colony defense [9], the neurophysiological basis remains unclear.

The mechanistic underpinnings of aggression in solitary animals have been studied. Biogenic amines, which are neuroactive chemicals, are involved in aggression in a number of animal species. In many arthropods including insects, octopamine (OA) is well known to be involved in aggression [10–13]. OA system is recently reported to involve in the variation of the aggression among castes (queens and workers) and sub-castes (major and miner workers) in ants [14, 15]. The disappearance of cooperative behavior in aggressive ant queens is also regulated by OA [16]. Tyramine (TA) is also considered to act as a true neuroactive chemical in invertebrates because of the presence of selective receptors and potentially purely TA neurons, although it was originally considered to be a biosynthetic precursor of OA and not itself a neuroactive chemical [11, 17–19].

In the present study, we focused on the biogenic amines, especially OA and TA, as candidate neuromodulators responsible for the higher aggressiveness of termite soldiers in the damp-wood termite *Hodotermopsis sjostedti* Holmgren. In the lower termites like *H*. *sjostedti*, older-instar larvae playing roles of workers have the potential to differentiate into other castes. Such individuals with a broad range of developmental options are currently termed pseudergates, originally defined as individuals without wing buds that develop regressively from nymphal instars [8, 20]. In *H*. *sjostedti*, soldiers are differentiated from pseudergates via presoldiers, and soldiers and pseudergates exhibit remarkable behavioral differences in colony defense under both natural and artificial conditions [21, 22]. To understand the neurophysiological underpinning of the caste specificity of defensive behavior, we first compared the levels of biogenic amines in the brain and suboesophageal ganglion (SOG) between the two castes. Because OA is related to the caste-specific high aggression in social hymenopterans, we further compared the localization and soma sizes of TA/OA neurons between soldiers and pseudergates. Finally, we performed pharmacological experiments to examine the effects of OA and TA application on defensive behavior in termites.

## Materials and Methods

### Insects

Colonies of *H*. *sjostedti* were sampled from decaying wood in evergreen forests on Yakushima Island in the Kagoshima Prefecture, Japan (30.3586° N, 130.5286° E). No specific permits were required for the field sampling and the locations are not privately-owned or protected in any way. The focal termite species is not endangered or protected species.

The colonies were maintained in the laboratory as stock at approximately 25°C under constant darkness. Pseudergates and soldiers from two independent colonies were used for high-performance liquid chromatography (HPLC) analysis. For immunohistochemistry, individuals from one colony were used. For TA and OA application, pseudergates from five and two independent colonies were used, respectively. To evaluate the anesthetizing effect on aggressiveness, we used pseudergates and soldiers from one colony. As in previous studies [22, 23], seventh-instar larvae were regarded as pseudergates.

### Measurement of biogenic-amine levels in the brain and SOG

First, we measured the levels of biogenic amines in the brain and SOG of soldiers and pseudergates. Taking termites from the wooden nest was usually associated with the disturbance of the colony. To minimize the effect on the biogenic-amine levels, we left them for several hours in 60-mm Petri dishes after the isolation, and then immediately froze them with cold spray. Termite brains and SOGs were dissected out in ice-cold sterilized saline (128.4 mmol/L NaCl, 2.7 mmol/L KCl, 1.8 mmol/L CaCl_2_, pH 6.7) in a dissecting dish cooled on ice. Dissected brains and SOGs were homogenized with a micro-glass homogenizer in 50 μl ice-cold 0.1 mol/L perchloric acid containing 12.5 ng/ml 3,4-dihydroxybenzylamine (DHBA) as an internal standard. Each sample was then transferred into a 1.5-ml micro tube, and centrifuged at 15,000 rpm for 30 min at 0°C. The supernatant was transferred to a micro-vial for analysis using a HPLC with electrochemical detection.

For simultaneous determination of the biogenic amines, we used the previously described method [24, 25] with minor modifications. The high-performance liquid chromatograph-electrochemical detection system comprised a solvent delivery pump (Ep-300, EICOM, Kyoto, Japan), a refrigerated automatic injector (231–401, Gilson Middleton, WI, USA), and a C_18_ reversed-phase column (250 mm x 4.6 mm id, 5 μm mean particle size, UG 120, Shiseido, Tokyo, Japan) maintained at 35°C in a column oven. An electrochemical detector (WE-GC, EICOM) with a glassy carbon electrode was set at 0.85 V against an Ag/AgCl reference electrode. The detector cell was maintained at 35°C by placing it in a column oven. Signals from the electrochemical detector were recorded and integrated using data analysis software (PowerChrom, ADInstrument, Castle Hill, NSW, Australia).

The mobile phase contained 0.8 mol/L monochloroacetic acid and 40 μmol/L of 2Na-EDTA adjusted to pH 3.6 with NaOH. Into this solution, 1.62 mmol/L sodium-1-octanesulfonate was added as the ion-pair reagent and 7% CH_3_CN was added as an organic modifier. The mobile phase buffer was filtered through a 0.22-μm filter (EMD Millipore, Billerica, MA, USA) and de-gassed by sonication. The flow rate was kept constant at 0.7 ml/min.

External standards were run before, midway through, and after the sample runs. External standards including *N*-acetyloctopamine, OA, DHBA, 3,4-dihydroxyphenylacetic acid, *N*-acetyldopamine, dopamine (DA), *N*-acetyltyramine, TA, *N*-acetyl-5-hydroxytryptamine, tryptophan, and 5-hydroxytryptamine (5-HT) were used to identify and quantify the biogenic amines. Each peak of a biogenic amine was identified based on the retention time and hydrodynamic voltamograms by comparing them to those of the standards. Measurements based on the peak amount of the chromatograms were obtained by calculating the ratio of the peak amount of the target substance to the peak amount of the external standard. Concentrations of the target substance were obtained by comparing these ratios between sample and standard chromatograms.

Because the sizes of SOG are different between soldiers and pseudergates in the focal species [26], we standardized the monoamine levels by the indexes for brain and SOG volumes. The indexes were calculated as (√((average of brain length)×(average of brain width)))^3 for brain-size index, and π/6×(average of SOG length)×(average of SOG width)×(average of SOG width) for SOG-size index. The length and width of brain and SOG were obtained from the original data of [26] (S1 Table). Then, the standardized levels were obtained by dividing the measured the absolute monoamine levels by the indexes (S2 Fig).

Statistical analysis was performed using R software (3.2.3) (http://www.r-project.org). Because the Shapiro-Wilk normality tests were significant in the several data categories (TA level in the soldier brain: W = 0.96, p = 0.5672, TA level in the pseudergate brain: W = 0.94, p = 0.1501, TA level in the soldier SOG: W = 0.94, p = 0.1563, TA level in the pseudergate SOG: W = 0.98, p = 0.8726, OA level in the soldier brain: W = 0.91, p = 0.04959, OA level in the pseudergate brain: W = 0.64, p < 0.0001, OA level in the soldier SOG: W = 0.92, p = 0.0638, OA level in the pseudergate SOG: W = 0.80, p = 0.0003, DA level in the soldier brain: W = 0.92, p = 0.0532, DA level in the pseudergate brain: W = 0.96, p = 0.3626, DA level in the soldier SOG: W = 0.95, p = 0.3366, DA level in the pseudergate SOG: W = 0.97, p = 0.5622, 5HT level in the soldier brain: W = 0.95, p = 0.268, 5HT level in the pseudergate brain: W = 0.96, p = 0.4447, 5HT level in the soldier SOG: W = 0.97, p = 0.6141, 5HT level in the pseudergate SOG: W = 0.98, p = 0.9013), the nonparametric exact Wilcoxon rank sum test was applied to all of the samples. The figure was prepared using the R package "ggplot2" (http://ggplot2.org).

### Immunostaining of TA and OA neurons

To compare the localization and soma size of TA and OA neurons between castes, we visualized the soma by immunohistochemical staining. Insects were anesthetized by cooling on ice. The brain-SOGs were dissected out and fixed in a fixative containing 1% glutaraldehyde in 0.1 mol/L sodium cacodylate buffer with sodium metabisulfite (SMB, Sigma-Aldrich, St. Louis, MO, USA) for 2 to 3 h in a refrigerator. After fixation, the preparations were treated with 0.5% sodium borohydride (NaBH_4_, Sigma-Aldrich) in 0.05 M Tris-HCL buffer containing 0.45% SMB (Tris-HCL SMB), pH 7.4, for 20 min to saturate double bonds. After washing with Tris-HCl SMB buffer containing 0.5% Triton X-100 (TX, Sigma-Aldrich; 15 min x 4, 30 min x 4), they were preincubated with 5% normal goat serum in Tris-HCl SMB buffer containing 0.5% Triton X-100 for 2.5 h. Then they were incubated with anti-TA antiserum (AB124, EMD Millipore; 1:200 in Tris-HCl SMB containing 0.5% TX, used in [19]) or anti-OA antiserum (kindly gifted from Sinakevitch IG, used in [27, 28], 1:200 in Tris-HCl SMB containing 0.5% TX) for three nights. These antibody specificities were revealed in [19, 28]. After washing in 0.05 mol/L in Tris-HCl buffer containing 0.5% TX, goat anti-rabbit Cy3-conjugated IgG (1:200 in 0.05 M Tris-HCl SMB containing 0.5% TX) was applied as the secondary antibody for two nights in a refrigerator. After the final wash in Tris-HCl buffer (15 min x 4, 30 min x 4), the preparations were dehydrated by increasing concentrations of EtOH and cleared in methyl salicylate. Each stained preparation was imaged frontally using a confocal imaging system (FV300, Olympus, Tokyo, Japan) with Uplan Apochromat ×10 (n.a. = 0.4) and ×20 (n.a. = 0.7) objectives. Neurons labeled with Cy3 were examined with 543 nm excitation and a long-pass emission filter (> 560 nm). Serial optical sections were acquired at 1.0- to 1.5-μm intervals throughout the entire depth of the stained neurons. All images were stored as TIF format files for later analysis. The signals of non-neural tissue (e.g. the remained fat bodies) were erased manually from the original images with FluoRender (http://www.sci.utah.edu/software/fluorender) for clarity. The maximum soma area of each neuron was measured using Image J software (http://rsb.info.nih.gov/ij/).

Statistical analysis was performed by R software. Because the Shapiro-Wilk normality tests revealed significant differences in the neuronal clusters, but not in the other clusters, the nonparametric exact Wilcoxon rank sum test and Student-t test were applied to each category, respectively. Because the soma size distributions of DUM1 and 2 showed clear bimodal patterns, we subcategorized these clusters by using k-means clustering method in R software. The larger neuronal clusters were named DUM1-L and DUM2-L, and smaller clusters were named DUM1-S and DUM2-S, and then applied to the statistical analysis. The histogram was prepared using the R package "ggplot2". The scheme of projection targets of TA/OA-l-ir neurons was drawn using Cytoscape.app (http://www.cytoscape.org/) and Illustrator CS6 software (Adobe Systems, San Jose, CA).

### Intracellular staining of DUM2-L neurons

For observation of DUM2-L neuronal projections, we performed intracellular staining with Lucifer Yellow, as previously described [29]. Cell bodies were visualized with a fixed-stage upright microscope (BX50WI or BX51WI; Olympus) equipped with DI differential interference contrast optics and long-working distance objectives (x40 LUMPlanFL/IR or x60 LUMPlanFL/IR water immersion), and a CCD camera (C2741-79; Hamamatsu Photonics) to enhance the contrast. Cell clusters on the brain surface could be clearly visualized with this system, allowing us to select cells for microelectrode insertion. In this study, we intended to stain DUM2-L neurons. Large cell bodies in the DUM cluster of the SOG were visualized and impaled with a microelectrode filled with Lucifer Yellow CH (4% in 0.1 mol/L LiCl_2_; Sigma-Aldrich). After impaling the soma, we iontophoretically injected the Lucifer Yellow using a 1 to 10 nA constant hyperpolarizing current for 3 to 5 minutes. This method efficiently stains the neurons. The obtained images were Z-stacked with maximal intensity using ImageJ software.

### Behavioral effect of TA and OA treatment

Pseudergates obtained from the stock colonies were used for the functional analyses of TA and OA. Because aggression of pseudergates is affected by the presence of other colony members [21], 20 to 30 pseudergates were kept overnight in Petri dishes lined with moistened filter paper before the experiments.

To evaluate the effect of OA or TA application on aggression, we utilized the same behavioral quantification system used in our previous report [21]. Individual termites were placed in a plastic Petri dish (diameter: 60 mm) lined with moistened filter paper. The dishes had an opening representing the main nest entrance and were placed in an arena (14 × 10 cm) in a plastic enclosure. OA-HCl (Sigma-Aldrich) and TA-HCl (Sigma-Aldrich) were dissolved in distilled water as 500 mmol/L stock solutions. Working solutions were prepared by diluting the stocks 1:5 in distilled water with red food coloring, yielding 100 mmol/L OA or TA. Experimental animals were anesthetized by 30-s exposure to CO_2_ in a 15-ml Falcon tube, and were injected with 0.5 μl into the base of the maxilla using a pulled glass capillary connected with plastic tube and microsyringe (1 mL), and then left in the Petri dish. The entrance was opened 45 to 60 min after the injection and an immobilized enemy ant (*Formica japonica*) was placed at the entrance. The termite behavior was recorded using a Digital Video Camera Recorder (Handycam DCR-PC5, Sony, Tokyo, Japan) for 3 min after encountering the enemy ant. Defensive behavior was defined as (i) biting: bite the ant, or (ii) orientation: stand facing the enemy ant. Because termite aggression varies under various meteorological conditions, such as humidity or unintended external stimuli, we performed the test and control (injection of distilled water containing red food coloring) experiments alternately. To evaluate the anesthetizing effect on aggressiveness, we performed the same experiment without the injection in intact soldiers and pseudergates. The aggression behavior was scored blindly to the treatments by the observer.

Statistical analysis was performed using R software. Because the Shapiro-Wilk normality tests were significant in several data categories (biting in the OA-control individuals: W = 0.72, p < 0.0001, biting in the OA-injected individuals: W = 0.86, p = 0.007, biting in the TA-control individuals: W = 0.84, p = 0.0004, biting in the TA-injected individuals: W = 0.89, p = 0.0041, biting in the uninjected soldiers: W = 0.85, p = 0.0576, biting in the uninjected pseudergates: W = 0.74, = 0.0024, orientation in the OA-control individuals: W = 0.97, p = 0.692, orientation in the OA-injected individuals: W = 0.98, p = 0.912, orientation in the TA-control individuals: W = 0.90, p = 0.0082, orientation in the TA-injected individuals: W = 0.85, p = 0.0006, orientation in the uninjected soldiers: W = 0.94, p = 0.5683, orientation in the uninjected pseudergates: W = 0.88, p = 0.1411), the nonparametric exact Wilcoxon rank sum test was applied to all of the samples. The figure was prepared using the R package "ggplot2".

## Results

### 1. Biogenic-amine levels in soldiers and pseudergates

**Fig 1:**
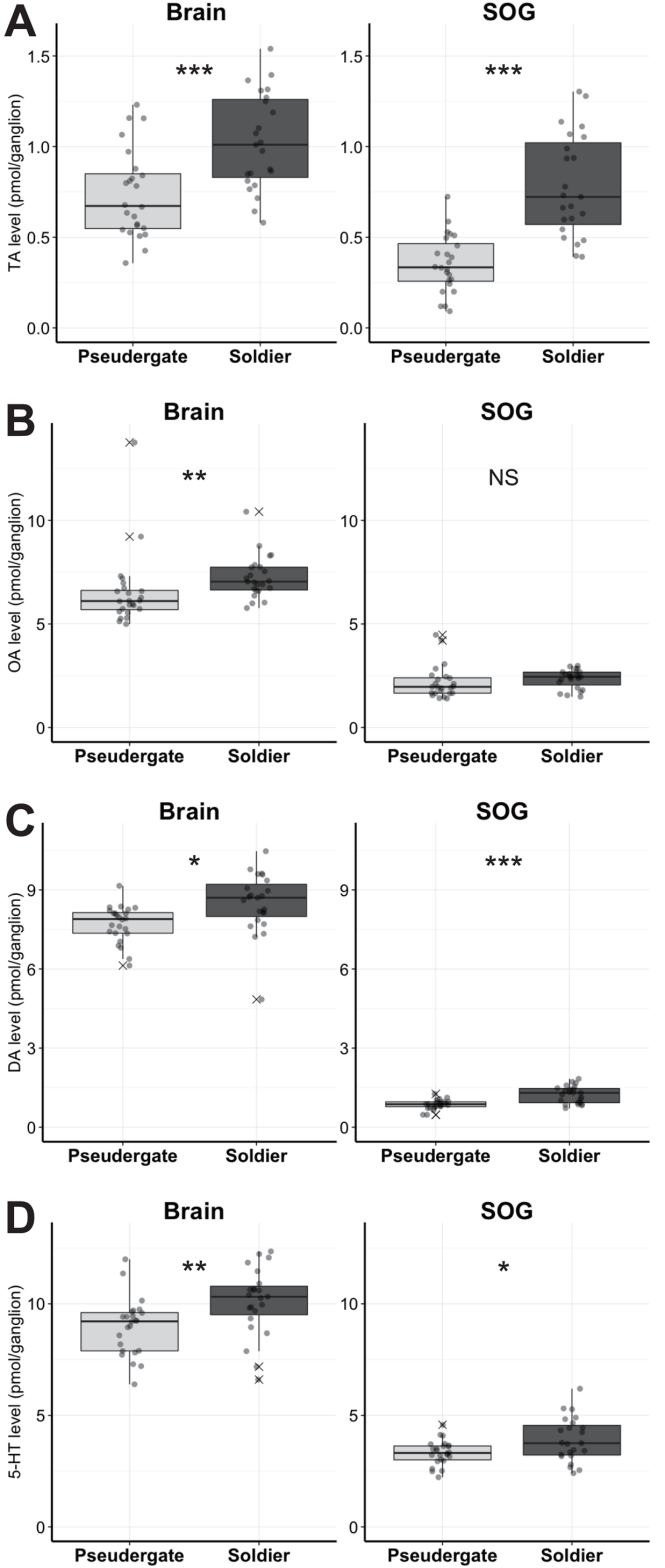
The absolute levels of biogenic amines in soldiers and pseudergates. Tyramine (A), octopamine (B), dopamine (C) and 5-HT (D) levels in pseudergates (light grey) and soldiers (dark grey) (N = 23 each). The circles indicate the aminergic levels in each termite individual. Box plots show median values (solid horizontal line), 50th percentile values (box outline), 90th percentile values (whiskers), and outlier values (crosses). Asterisks indicate statistical significance (***p < 0.001, **p < 0.01, *p < 0.05 in exact Wilcoxon rank sum test).

We first compared the amount of biogenic amines in brain and SOG between soldiers and pseudergates. In brain, the absolute levels of all biogenic amines were significantly higher in soldiers than in pseudergates (Fig 1, Table 1 and S1 Fig, TA: W = 111, p = 0.0003; OA: W = 118, p = 0.0005; DA: W = 131, p = 0.0016; 5-HT: W = 136, p = 0.0024, Exact Wilcoxon Rank Sum Test). Because the brain sizes are not significantly different between soldiers and pseudergates in the focal termite species [26], the result indicated the brain levels of these biogenic amines were higher in soldiers than in pseudergates. In SOG, the absolute levels of TA, DA, 5-HT were significantly higher in soldiers than in pseudergates (Fig 1, Table 1, TA: W = 43, p < 0.0001; OA: W = 189, p = 0.0653; DA: W = 98, p < 0.0001; 5-HT: W = 175, p = 0.0315, Exact Wilcoxon Rank Sum Test). In contrast to brain, the size of SOG is larger in soldiers than in pseudergates [26]. Therefore, we standardized the biogenic amine levels by the estimated ganglion-volumes indexes calculated with the length and width of the ganglia (S2 Fig). Among TA, DA, and 5-HT, only the TA level was still significantly higher in soldiers even when normalized by the SOG volume (Table 1, S2 Fig, brain: W = 107, p = 0.0002, SOG: W = 143, p = 0.0041, Exact Wilcoxon Rank Sum Test), suggesting that the TA level in SOG is higher in soldiers than that in pseudergates.

**Table 1:** Comparison of biogenic-amine levels in brain and SOG.

Amine	Ganglion	Mean (absolute)		SD (absolute)		Mean (standardized)		SD (standardized)	
		Soldier	Pseudergate	Soldier	Pseudergate	Soldier	Pseudergate	Soldier	Pseudergate
5-HT	Brain	10.100	8.961	1.512	1.279	10.100	8.768	1.512	1.251
5-HT	SOG	3.917	3.321	0.989	0.550	3.917	4.965	0.989	0.823
DA	Brain	8.495	7.707	1.153	0.697	8.495	7.540	1.153	0.682
DA	SOG	1.239	0.861	0.325	0.182	1.239	1.286	0.325	0.272
OA	Brain	7.258	6.521	1.031	1.783	7.258	6.380	1.031	1.745
OA	SOG	2.346	2.185	0.455	0.793	2.346	3.267	0.455	1.186
TA	Brain	1.024	0.736	0.265	0.243	1.024	0.720	0.265	0.238
TA	SOG	0.782	0.353	0.282	0.158	0.782	0.528	0.282	0.236

### 2. Comparison of the localization and soma size of TA- and OA-like immunoreactive neurons between soldiers and pseudergates

#### 2.1 TA-like and OA-like immunoreactive neurons

**Fig 2:**
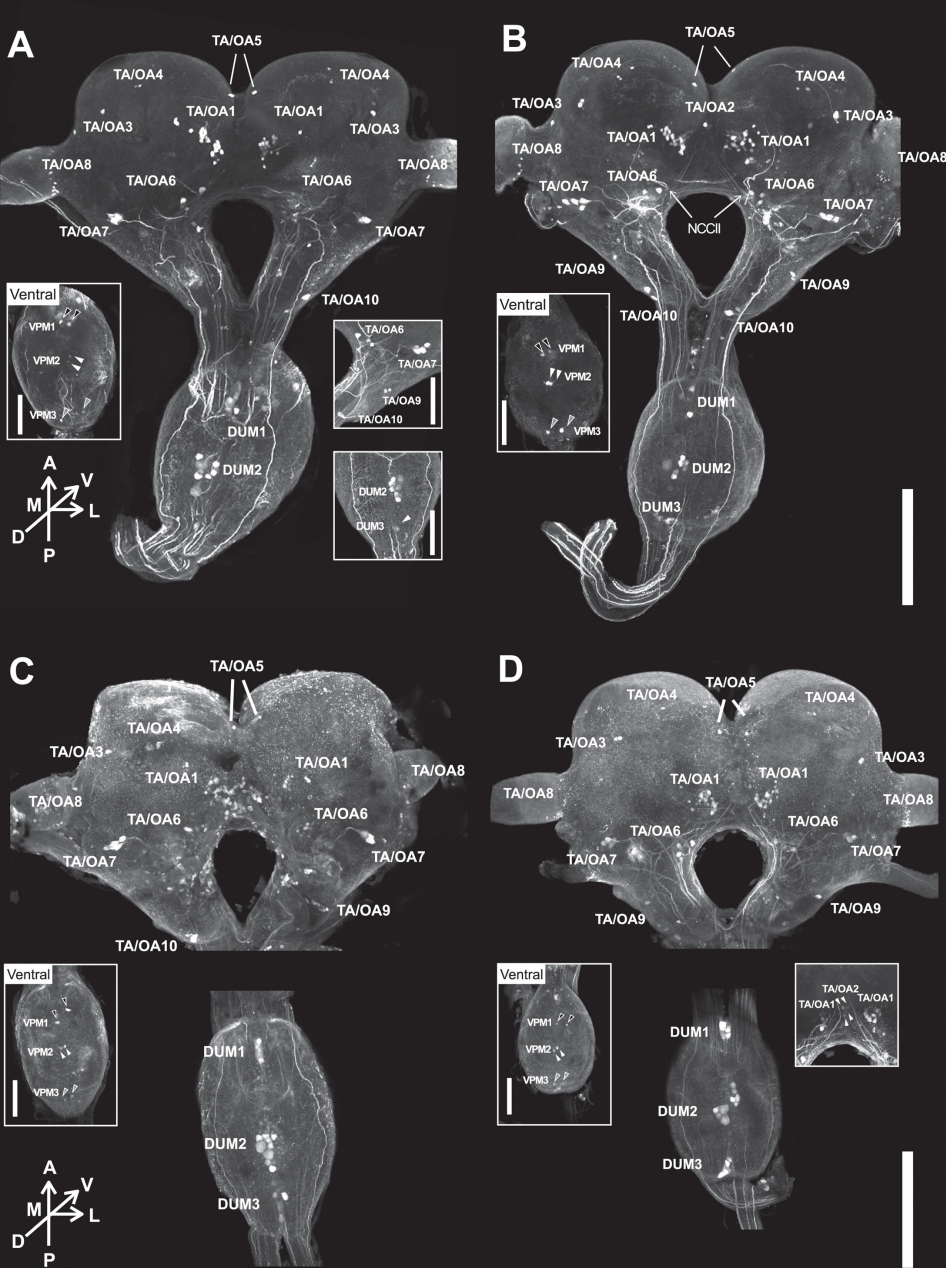
Localization of TA-like-immunoreactive (TA-l-ir) and OA-like-immunoreactive (OA-l-ir) neurons. TA-l-ir neurons in soldiers (A) and pseudergates (B). OA-l-ir neurons in soldiers (C) and pseudergates (D). Left insets indicate the ventral view of SOG. VPM1, 2, 3 are indicated with black, white, grey filled arrowheads, respectively. Right insets indicate images of other individuals. In right inset of (A), white arrowhead is DUM3 neurons. In right inset of (D), white arrowhead is TA/OA2 neurons. Scale bars indicate 500μm in main picture, and 250μm in insets.

**Fig 3:**
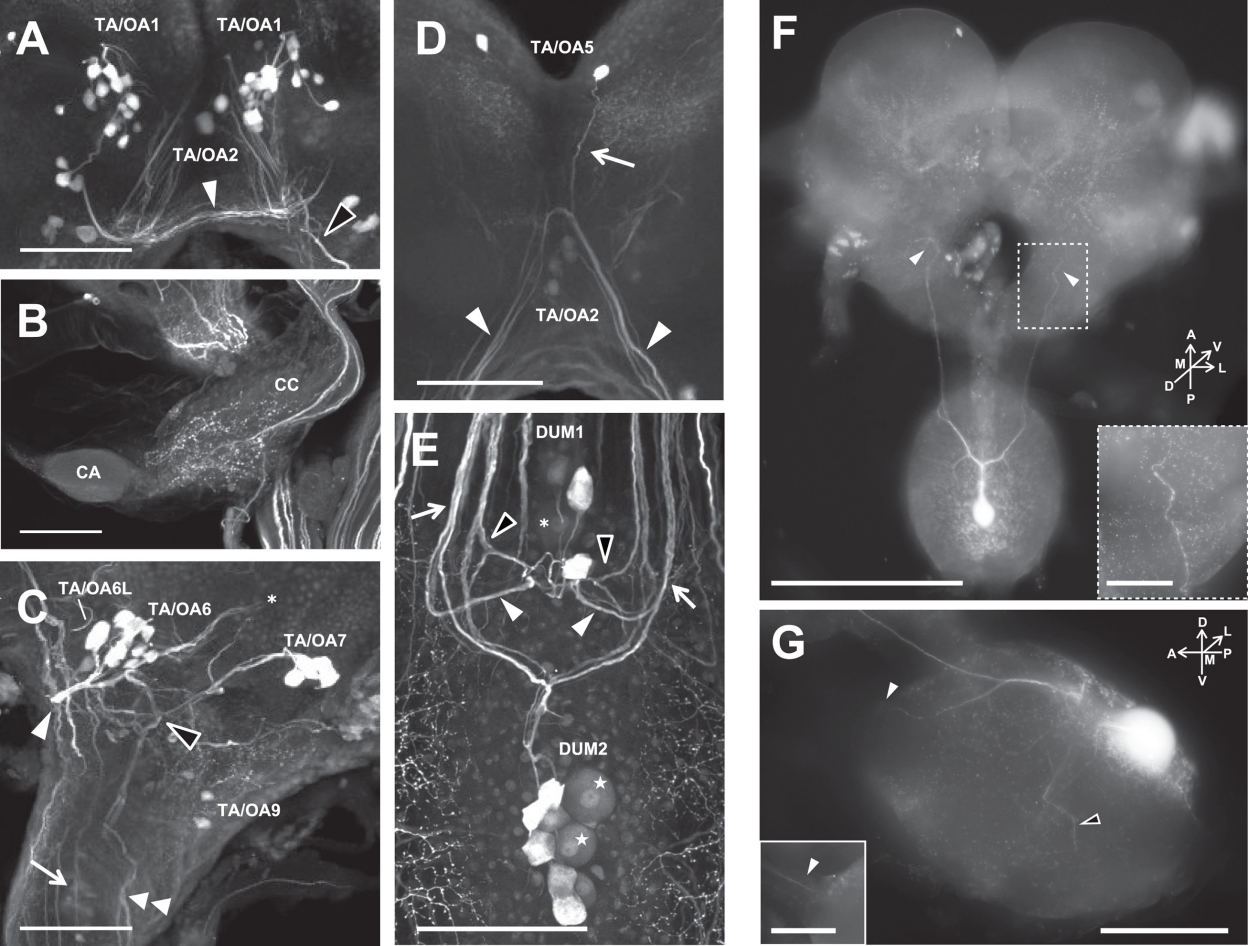
Projection pattern of TA-l-ir neurons. (A~E) Immunostaining using an anti-TA antibody identified single neurons or clusters of immunostained neurons and their fibers. (A) TA/OA1 cluster forms the commissure under the central complex. (B) The corpora cardiaca (CC) is innervated by TA/OA3. Corpora allata (CA). (C) TA/OA6 and TA/OA7 show characteristic projection patterns. (D) TA/OA2 and TA/OA5 share the descending bundle. (E) In the SOG, DUM1 and DUM2 clusters show symmetrical projection patterns with different staining intensities. Scale bars indicate 100 μm. (F, G) Projection pattern of a DUM2-L neuron visualized by intracellular staining. DUM2-L neurons projected mainly into the mandibular nerves and tritocerebrum, and send thin neurites into the maxillary segment of the SOG. (F) Dorsal view of a DUM2-L neuron. Numerous varicosities were observed in the tritocerebrum (arrowheads). Inset indicates the magnified image of the DUM2-L projection area in tritocerebrum. Scale bars indicate 500μm in main picture, and 100μm in insets. (G) Lateral view of a DUM2-L neuron in the SOG. The neurite extends to the mandibular nerve (white arrowheads) and maxillary neuromere (a black arrowhead). Inset is the image of the different depthplane, indicating the DUM2-L projection into the mandibular nerve. Scale bars indicate 200μm in main picture, and 100μm in insets.

Because the OA was known to be involved in the aggressive variation among castes in social hymenopterans [14–16, 30, 31], we further investigated the neurons contributing the higher TA and OA levels in soldiers and identified the candidate neuroanatomical foci regulated by these neurons, by comparing the localization, and soma sizes of TA- and OA-like immunoreactive (TA-l-ir and OA-l-ir) neurons between the two castes. TA immunostaining revealed over 70 somata in the brain and SOG (Figs 2 and 3, Tables 2 and 3, S1 Movie). Although the staining level was relatively weak, similar sets of neuronal clusters were also visualized by OA immunostaining (Fig 2, Tables 2 and 3). Therefore, we could not determine whether these neurons release OA or TA under natural conditions. Since TA-l-ir and OA-l-ir neurons in termites were not described so far, we termed the neuronal populations according to the soma localization and projection patterns, as stated below. All of the TA-l-ir neuronal clusters in SOGs were identified as counterparts of dorsal unpaired median (DUM 1, 2, and 3) and ventral paired median (VPM 1, 2, and 3) neuron clusters observed in various insect species (Table 2) [19, 27, 32]. In the brain, the corresponding clusters were not clear, and were thus classified as TA/OA1–10 clusters according to the localization (Figs 2 and 3, Tables 2 and 3, S1 Movie).

**Table 2:** Comparison of TA/OA-l-ir neuronal clusters of termite, locust (1, 2) and cockroach (3).

Termite	Locust^a^	Locust^b^	Cockroach^c^
TA/OA1	C7	TA3 (> 10, 20–25μm)	G1
TA/OA2	-	-	-
TA/OA3	-	TA4? (2 pairs, 15–20μm)	a part of G0? (14 pairs)
TA/OA4	-	-	a part of G0? (14 pairs)
TA/OA5	-	-	-
TA/OA6	C1 (6–8 pairs)	OA1/TA (7 pairs, 20–60μm)	G3a,b (13 pairs)
TA/OA7	-	OA3/TA	G5
TA/OA8	-	TA6	Not named
TA/OA9	-	-	-
TA/OA10	C2	OA2/TA (1 pair, 20–60μm)	-
DUM1	-	-	DUM1 (5 neurons, 40μm)
DUM2	-	-	DUM2 (2 and 4 neurons, 60μm and 40μm)
DUM3	-	-	DUM3 (6 neurons, 40μm)
VPM1	-	-	-
VPM2	-	-	-
VPM3	-	-	-

^a^ Stevenson and Sporhase-Eichmann [32]

^b^ Kononenko, Wolfenberg [19]

^c^ Sinakevitch, Niwa [27]

**Table 3:** Statistical comparison of the TA/OA-l-ir-soma sizes between soldiers and pseudergates (see also Fig 2).

Cluster	Sum of cell num (N = 7 ind)		Cell num in an individual		Mean size of soma (μm^2^)		Min size of soma (μm^2^)		Max size of soma (μm^2^)		Shapiro-Wilk normality test (p)		Student-t test (p)	Wilcoxon rank sum test (p)
	Soldier	Pseudergate	Soldier	Pseudergate	Soldier	Pseudergate	Soldier	Pseudergate	Soldier	Pseudergate	Soldier	Pseudergate		
TA/OA1	143	133	19–22 pairs	16–22 pairs	90.426	94.117	35.556	33.420	197.333	267.018	**<0.001**	**<0.001**	-	0.872
TA/OA2	8	13	2 pairs	2 pairs	109.390	139.039	88.417	113.794	130.163	166.223	0.934	0.340	**0.001**	-
TA/OA3	14	14	2 pairs	2 pairs	147.779	141.928	112.000	93.225	181.333	210.432	0.103	0.226	0.600	-
TA/OA4	23	25	3–5 pairs	3–5 pairs	61.047	55.804	42.215	38.555	89.285	79.139	0.122	0.418	0.128	-
TA/OA5	7	7	1 pair	1 pair	145.829	119.873	123.783	90.185	181.173	167.111	0.149	0.544	**0.074**	-
TA/OA6	54	50	7–10 pairs	6–8 pairs	158.487	148.155	47.745	70.359	364.277	280.030	**0.001**	**0.003**	-	0.515
TA/OA7	30	30	4–5 pairs	4–5 pairs	276.652	247.427	142.222	172.379	416.000	371.344	0.318	0.130	**0.044**	-
TA/OA8	70	100	7–15 pairs	11–18 pairs	23.354	21.648	10.610	8.842	44.444	58.847	**0.030**	**<0.001**	-	**0.005**
TA/OA9	19	14	2–4 pairs	2 pairs	112.020	132.261	58.355	72.502	179.556	204.040	0.445	0.278	0.163	-
TA/OA10	12	14	2 pairs	2 pairs	192.146	184.213	112.000	60.123	279.111	373.374	0.547	0.746	0.777	-
VPM1	2	7	1 pair	1 pair	239.610	95.376	231.652	76.038	247.567	136.162	NA	0.079	**0.001**	-
VPM2	10	9	1 pair	1 pair	148.325	128.232	91.953	92.444	204.444	184.092	0.752	0.468	0.180	-
VPM3	14	14	1 pair	1 pair	169.562	175.957	128.000	127.320	208.663	250.222	0.709	0.534	0.591	-
DUM1-S	27	27	2–5 neurons	2–5 neurons	214.649	181.462	95.490	111.405	321.778	273.456	0.495	0.151	**0.011**	-
DUM1-L	6	10	1–2 neurons	1–2 neurons	612.650	390.485	538.243	295.312	710.622	511.049	0.404	0.947	**<0.001**	-
DUM2-S	48	48	7 neurons	7 neurons	221.626	190.096	138.667	125.552	330.678	336.012	0.672	0.980	**<0.001**	-
DUM2-L	14	13	2 neurons	2 neurons	684.015	576.328	578.245	456.230	862.222	707.770	0.336	**0.017**	-	**<0.001**
DUM3	8	20	1–3 neurons	4–7 neurons	268.849	209.928	208.663	87.578	350.034	303.841	0.704	0.515	**0.009**	-

TA/OA1 cluster: The TA/OA1 cluster is a group of 14 to 22 pairs of somata with various diameters (10–20 μm) located at the ventromedial surface of the protocerebrum (Figs 2 and 3A, Tables 2 and 3, S1 Movie). Neurites project contralaterally and form the commissure under the central complex (Fig 3A, white arrowhead, S1 Movie). Localization of the soma cluster is similar to that of the OA-l-ir G1 cluster in the cockroach and the TA3 cluster in the locust [19, 27].

TA/OA2 cluster: Two pairs of somata (≈12 μm diameter) located anteroventral to the central complex (Figs 2, 3A and 3D, Tables 2 and 3, S1 Movie). The axons run bifurcately into both circumesophageal connectives and reach the mandibular segments in the SOG (Fig 3D, white arrowheads, S1 Movie). They seem to form bundles with TA/OA5 clusters (Fig 3D, arrow).

TA/OA3 cluster: Two pairs of neurons located at the dorsolateral part of protocerebrum (Fig 2, Tables 2 and 3, S1 Movie), and projected into the corpora cardiaca ipsilaterally through the NCCII (Fig 2B, white arrow). In the corpora cardiaca, numerous varicosities were observed (Fig 3B). In addition, the neurons projected into the anterior regions of the central complex.

TA/OA4 cluster: The cluster contained 3 to 5 small somata (< 10 μm diameter) in the dorsal region of the protocerebrum on each side, as previously reported in the cockroach and locust (G0 cluster in cockroach and T4 cluster in locust, [19, 27]), although the projections were poorly matched (Fig 2, Tables 2 and 3, S1 Movie). The branching pattern was not resolved in this study.

TA/OA5 neurons: A single pair of somata located at the anteromedial part of the protocerebrum (Figs 2 and 3D, Tables 2 and 3, S1 Movie). Neurites of the TA/OA5 and TA/OA2 somata seemed to fuse and project into the mandibular segments of the SOG (Fig 3D, arrow).

TA/OA6 cluster: The TA/OA6 cluster included 3 to 10 somata of various sizes (Figs 2 and 3C, Tables 2 and 3, S1 Movie, 10–20 μm in diameter). A single pair of relatively large somata (TA/OA6-L) extended their neurites straight into the ipsilateral circumesophageal connectives (Fig 3C, arrow), and then bifurcated in the mandibular segment of the SOG. One of the branches extended into the maxillary segments, while the other projected across the midline of the SOG to join the counterpart neurites. The projections of other neurons (TA/OA6-S) formed a bundle and extended medially (Fig 3C, white arrowhead), and then turned dorsolaterally to pass over the dorsal surface of the protocerebrum (Fig 3C, asterisk). The localization and projection pattern of the TA/OA6 cluster were similar to those of the OA-l-ir G3 cluster in the cockroach and the OA1/TA cluster in the locust [19, 27].

TA/OA7 cluster: The TA/OA7 cluster comprised 4 to 5 pairs of somata with a large diameter (20–30 μm) located anterolaterally in relation to the antennal lobe region (Figs 2 and 3C, Tables 2 and 3, S1 Movie). The projections of the neurons diverged dichotomously in the posterior part of the deutocerebrum (Fig 3C, black arrowhead). One neuronal branch bifurcated again into the tritocerebrum, and both projections ran straight into the thoracic and/or abdominal ganglia through the SOG (Fig 3C, double arrowhead). Another branch extended to the medial deutocerebrum, including the region above the central complex (Fig 3A, black arrowhead). Due to the similarity of the soma locations and branch patterns, we considered this to be homologous to the G5 cluster in the cockroach and the OA3/TA cluster in the locust [19, 27].

TA/OA8 cluster: In the lobula, numerous (7–18) small somata (≈ 5 μm in diameter) were observed (Fig 2, Tables 2 and 3, S1 Movie).

TA/OA9 neurons: The TA/OA9 cluster contained 2 to 5 pairs of somata located in the lateral cell layer of the tritocerebrum (Fig 2, Tables 2 and 3, S1 Movie). A pair of the somata projected into the contralateral tritocerebrum through circumesophageal connectives.

TA/OA10 neurons: Two pairs of somata were located posterolaterally in the tritocerebrum (Fig 2, Tables 2 and 3, S1 Movie). A pair with large soma (≈ 25 μm in soma diameter) projected anteriorly. The branching pattern of a smaller pair was not observed in this study. The cell localizations of the larger somata were similar to those of the OA2/TA cluster in the locust [19].

**Fig 4:**
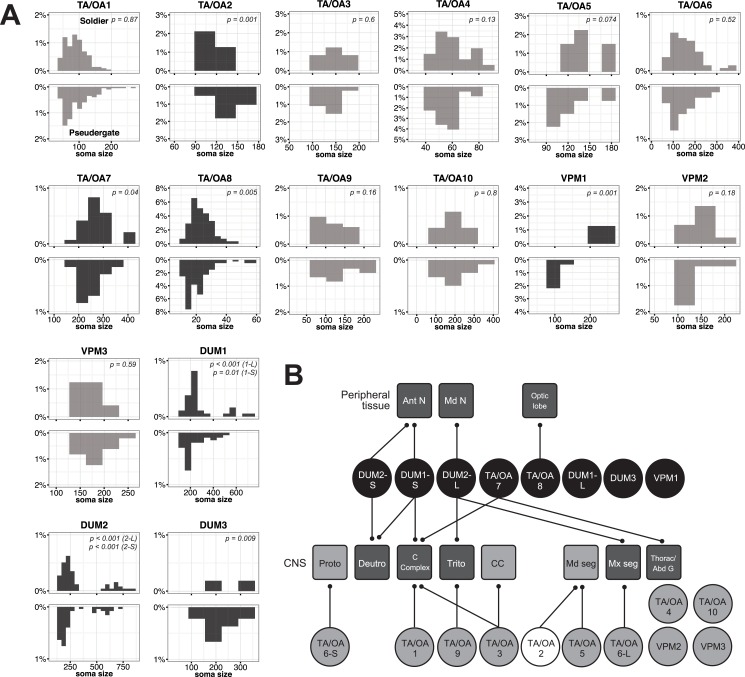
Comparison of soma size distributions of TA/OA-l-ir neurons between soldiers and pseudergates. (A) Each upper and lower panel indicates the soma size distribution of TA/OA-l-ir neurons in soldiers and pseudergates in the neuronal cluster, respectively (See also Table 3). Dark grey histograms indicate statistical differences between castes. (B) Projection targets of TA/OA-l-ir neurons. The TA/OA neuronal clusters and projection target are indicated as circles and rounded squares, respectively. Neuronal clusters that are soldier- and pseudergate-specifically enlarged are coloured black and white, respectively. Light grey fill indicates no significant differences in the soma sizes between castes. Target regions are subdivided as antennal nerve (Ant N), mandibular nerve (Md N), optic lobe, protocerebrum (Proto), deutocerebrum (Deuto), central complex (C Complex), tritocerebrum (Trito), corpora cardiaca (CC), mandibular segment of SOG (Md seg), maxillary segment of SOG (Mx seg), and thoracic and abdominal ganglia (Thorac/Abd G). The target regions of the soldier-enlarged neurons are filled in dark grey.

DUM1 clusters: The DUM1 cluster comprised 3 to 7 somata located on the most posterior part of the SOG (Figs 2 and 3E, Tables 2 and 3, S1 Movie). The soma sizes of 1 or 2 neurons (DUM1-L) were large (> 25 μm in diameter, Fig 3E asterisk), while the rest (DUM1-S) were relatively small (< 20 μm in diameter), as the size distribution of these neurons showed a bimodal pattern (Fig 4A). Because TA-l-ir of the DUM-L neurons was constantly weaker than that of other neurons (Fig 3E, asterisk), the projection patterns were not clear. The axons of DUM1-S neurons extended posteriorly along the midline, and then symmetrically projected into various regions of the brain and SOG (Fig 3E). The bundle on each side was divided into two branches, and posterior branch projected into the antennal nerve through the circumesophageal connective (Fig 3E, white arrowheads). The anterior branch bifurcated again (Fig 3E, black arrowheads), with one branch running posteriorly and the other entering into the posterior deutocerebrum and the bottom part of the central complex.

DUM2 clusters: The DUM2 cluster contained nine somata on the dorsomedial part of the maxillary segment of the SOG (Figs 2, 3E–3G, Tables 2 and 3, S1 Movie). The neurons were subdivided into two groups; 2 large neurons (DUM2-L neurons, 25–35 μm in soma diameter, Fig 2E indicated by stars) and 7 small neurons (DUM2-S neurons, ≈ 20 μm in soma diameter). The staining intensity of DUM2-L neurons was consistently low (Fig 3E). Among the 7 small neurons, 4 somata constantly showed strong TA-like immunoreactivity, while 3 were stained weakly. Intracellular staining with Lucifer-yellow revealed that DUM2-L neurons projected mainly into the mandibular nerves and tritocerebrum, and sent thin neurites into the maxillary segment of the SOG (Fig 3F and 3G). In the tritocerebrum, numerous varicosities were observed (Fig 3F, inset). DUM2-S projections fused with the tracts of DUM1 neurons and entered into the antennal nerves and posterior deutocerebrum (Fig 3E, white arrows).

DUM3 clusters: The DUM3 cluster included 1 to 7 somata on the dorsoposterior part of the SOG (Fig 2, Tables 2 and 3, S1 Movie). Some of the primary axons extended anteriorly, but the terminals could not be followed.

VPM1-3 clusters: A single pair of cells was located at the ventromedial part of each of the three segments of the SOG (Fig 2, Tables 2 and 3, S1 Movie).

### 2.2 Comparison of TA/OA-l-ir neurons between soldiers and pseudergates

When comparing soldiers and pseudergates, the similar neuronal populations were labeled by TA and OA immunostaining (Fig 2, Table 3), indicating that soldiers and pseudergates possess the same set of TA/OA-l-ir neurons. On the other hands, comparison of soma sizes revealed a significant difference between the castes (Fig 4, Table 3). Interestingly, not all of the TA/OA-l-ir neuronal somata differed between the castes. Soma size of TA/OA1, TA/OA3, TA/OA4, TA/OA5, TA/OA6, TA/OA9, TA/OA10, VPM2 and VPM3 clusters were not significantly different between the castes, while somata of TA/OA7, TA/OA8, VPM1, DUM1, DUM2 and DUM3 clusters were selectively larger in soldiers, and somata of TA/OA2 clusters were selectively larger in pseudergates (Fig 4A, Table 3). As stated above, DUM1 and DUM2 clusters contain two different-sized sub-populations (DUM1-L and DUM1-S, or DUM2-L and DUM2-S). Somata of all these DUM neuronal sub-populations in soldiers were larger than those in pseudergates (Table 3). The projections of the enlarged TA/OA-l-ir neurons extended to various target regions, including the central complex, SOG, thoracic and/or abdominal ganglia, and antennal, mandibular, maxillary nerves (Fig 4B). These projection targets of the enlarged TA/OA-l-ir neurons might have important roles in soldiers.

### 3. Behavioral effects of OA and TA treatment

**Fig 5:**
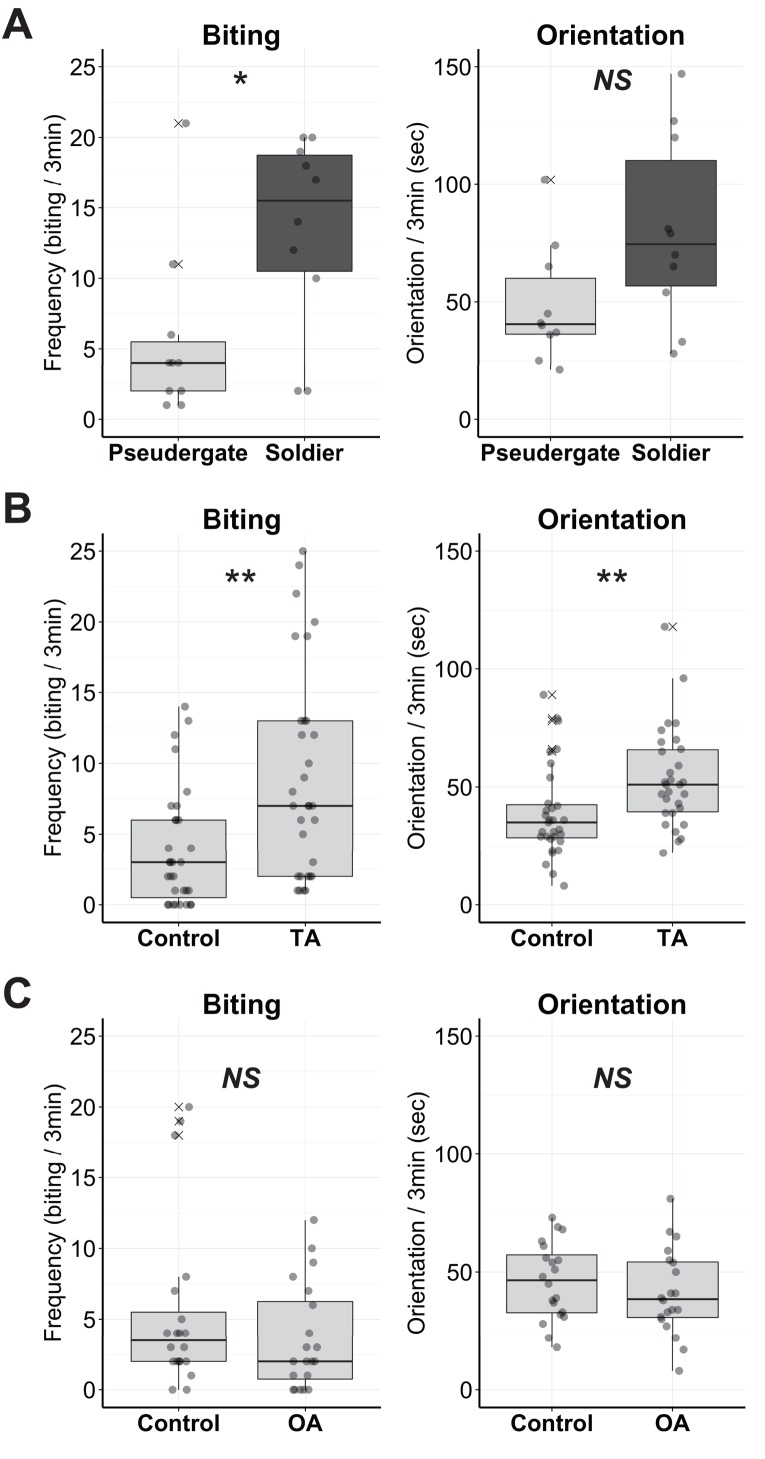
Functional analyses of TA and OA application in pseudergates. (A) Defensive behavior of soldiers and pseudergates anaesthetised but uninjected as controls (N = 10 each). (B) Defensive behavior of TA-treated pseudergates and controls (N = 31 each). (C) Defensive behavior of OA-treated pseudergates and controls (N = 20 each). The circles indicate the biting frequency or duration of orientation in each termite individual. Box plots show median values (solid horizontal line), 50th percentile values (box outline), 90th percentile values (whiskers), and outlier values (crosses). Asterisks indicate statistical significance (**p < 0.01, *p < 0.05 in exact Wilcoxon rank sum test).

To reveal the behavioral function of OA and TA, the aggression levels of the OA- or TA-treated termites were observed. As an indicator of aggression level, defensive behaviors (i.e., biting and orientation) toward a predatory ant species, *Formica japonica*, were quantified. The behavioral quantification of uninjected but anaesthetised individuals confirmed that soldiers exhibited higher aggressiveness than pseudergates (Fig 5A, biting: W = 77, p = 0.0404, orientation: W = 74.5, p = 0.0660, Exact Wilcoxon Rank Sum Test), although biting frequencies were decreased compared with unanaesthetized termites [21]. After TA treatment, pseudergates exhibited a significantly higher level of aggression than controls (Fig 5B, W = 258.5, biting: p = 0.0014, orientation: W = 262.5, p = 0.0018, Exact Wilcoxon Rank Sum Test). Pseudergates often showed continuous biting behavior even when not in direct contact with the ants (data not shown). Unlike TA, injection of OA had no detectable effects on defensive behavior (Fig 5C, biting: W = 240, p = 0.390, orientation: W = 232.5, p = 0.281, Exact Wilcoxon Rank Sum Test).

## Discussion

The present study revealed that TA/OA systems potentially responsible for the defensive division of labor in the damp-wood termite *H*. *sjostedti*. Although the caste differences in aminergic systems have already been documented in several social hymenopterans [33–36], this is the first report suggesting that the aminergic modulation also functions in social behavior in non-hymenopteran social insects.

In soldier termites, the TA and OA levels in brains and TA level in SOG were significantly increased. Consistent with this, the TA and OA immununostaining revealed that the particular TA/OA-l-ir neuronal somata were specifically enlarged in the termite soldiers. The soma sizes of particular TA/OA-l-ir neuronal clusters, i.e., TA/OA2, TA/OA7, TA/OA8, DUM1, DUM2, DUM3, and VPM1, differed significantly between soldiers and pseudergates. Not all TA/OA-l-ir neurons are enlarged in soldiers, suggesting that this size differences are not only due to differences in the soma enlargement of whole neuronal population. The biological function of the soma enlargement remains unclear, but given that most protein synthesis generally occurs in the cell body, the enlargement might relate to increased synthesis of neurotransmitter-related enzymes, cytoskeletal proteins, and other proteins, to maintain caste-specific neuronal networks. Mandibular motor neurons (MdMNs) used while attacking enemies also enlarge during the course of soldier differentiation in the focal species [26], and thus the enlargement of TA/OA-l-ir neurons likely occurs simultaneously in the developmental process. In this species, several genes that are possibly involved in axonal growth or neural migration are upregulated in the course of soldier differentiation [37], suggesting that these genes play some role in the soldier-specific development of TA/OA neurons and MdMNs.

Visualization of these neurons by TA-like immunostaining revealed candidate neuroanatomical foci regulated by the soldier-specific enlarged neurons. Because OA-like immunostaining visualized the similar sets of neurons, we could not determine whether TA or OA is the major neuromodulator in the enlarged neurons. Among the observed TA-l-ir neuronal clusters, the soma sizes of the DUM clusters were most significantly increased in the soldiers. OA immunostaining labeled similar DUM neurons in the termite SOG, as is the case in the locust, in which DUM neurons in the SOG are co-labeled with anti-TA and anti-OA antibodies [19]. Although DUM neurons generally secrete OA to the targets, a recent study indicated that some of them also secrete TA upon electrical stimulation [38, 39]. Unlike the thoracic and abdominal ganglia, DUM neurons in the SOG project to the brain as well as to peripheral effecter organs, and are considered to be the most important source of OA and TA in the insect brain [11]. Also in termites, DUM neurons in the SOG extend projections to both peripheral tissues (antennal nerve and mandibular nerve) and the brain (central complex and posterior deutocerebrum). In the periphery, OA modulates various sensory systems, including tactile systems in the antennae [40]. Modulation of the mechanosensory systems might be involved in the increased sensitivity to air movement and vibration of the termite soldier, when the colony is invaded. On skeletal muscles in *Drosophila*, OA and TA have opposite functions; OA enhances the amplitude of neutrally evoked excitatory junction potentials and TA reduces it [18, 41]. Because DUM2-L neurons innervate to the mandibular nerves, and the mandibular muscles are larger in termite soldiers than in pseudergates, enlargement of the efferent DUM neurons may reflect the larger area of the target muscle. DUM1-S neurons also project into the central complex and posterior deutocerebrum in the brain. The central complex plays a key role in locomotor control [42]. During predatory invasion, workers (pseudergates and larvae) and soldiers exhibit a different locomotor pattern; workers immediately escape deep into the nest, while soldiers remain at the entry pathway and prepare for counterattack (“orientation” shown in this study) [21, 43]. Although the physiological role of OA and TA in the central complex remains unknown in any insect species, modulation of the target brain regions might leads to changes in locomotor pattern and aggressiveness in soldiers during predator invasion. In brain, the soma sizes of the TA/OA7, and TA/OA8 clusters were larger in soldiers than in pseudergates. These projection areas may also have important roles in the higher aggressiveness and defensive behavior of the termite soldiers. Interestingly, the locust homologous cluster of TA/OA7 alters OA-like immunoreactivity in response to stressful stimuli including loud noises [19], thus TA/OA7 clusters in termites may play some role in the behavioral change in response to the air vibration caused by invasion of enemies and warning of nestmates. The TA/OA8 cluster neurons are located in the optic lobe. Although compound eyes of soldiers and workers (larvae and pseudergates) are underdeveloped compared to winged reproductives, both of them exhibit negative phototaxis, indicating that they are able to detect light [44]. Especially in soldier, the light may act as a factor inducing their defensive reaction because they show a defensive posture with their head pointing towards the lighted region [45]. Recently, phototaxis in honeybee were shown to be negatively and positively modulated by OA and TA, respectively [46]. Although the caste difference of light sensitivity in termites was previously unreported, the enlargement of TA/OA8 neurons in soldiers might be related to the regulation of light sensitivity related to the colony defense.

Pharmacological application of TA to individual termites induced increased defensive behavior toward the predators, while OA application at the same concentration had no detectable effect. Because TA is a functional neuromodulator and a precursor of OA, and several OA receptors are known to cross-react with TA (reviewed in Lange (18)), it is difficult to conclude that the behavioral effect of TA treatment is due to a neuromodulatory function of TA or the indirect effect increasing the synthesis and release of OA. Together with the results of aminergic levels and immunostaining, the pharmacological results suggest that the higher TA and/or OA cause the high aggressiveness and defensive behavior of soldiers. The fact that OA also causes behavioral variations among/within castes in social hymenopterans [14–16, 30, 31], the physiological mechanisms may be repeatedly co-opted in the evolutionary acquisition of social behavior of various insect lineages.

Generally, the biogenic amine levels in animals are affected by environmental conditions, including social interactions. In social hymenopterans, biogenic amine levels are affected by the presence of other castes [35, 47–51]. We previously reported that aggression in pseudergates changes depending on the social context, potentially resulting in the rapid and flexible responses for colony defense [21]. Such behavioral plasticity may also be explained by function of TA and OA; for example, the presence of reproductives may increase TA/OA levels, resulting in higher aggressiveness of pseudergates, while the presence of soldiers may decrease these levels, resulting in lower aggressiveness of them. In this study, we only analyzed the baseline levels of TA and OA in the isolated soldiers and pseudergates. Further investigations of the social effect on the aminergic levels will reveal the neurophysiological mechanisms of social context-dependent aggression and flexible defense strategy in termites. Moreover, considering that biogenic amines generally interact with some endocrine factors such as juvenile hormone, which is involved in termite soldier differentiation [52–56], the changes in TA/OA levels could induce subsequent physiological alterations that lead to morphological caste differentiation. Future studies of the interactions of biogenic amines and endocrine factors will provide insight into the molecular underpinnings of task allocation in termites.

Other than TA and OA, the levels of 5-HT and DA are also increased in soldier brains, although the responsible neurons and behavioral function were not investigated in this study. 5-HT is known to modulate aggression in ants and fruit flies [57–61]. In American cockroaches (*Periplaneta americana*), species closely related to termites, the application of 5-HT suppresses the escape circuit [62]. Although DA is well-known to be involved in reproductive regulation of social hymenopterans [47, 49, 51, 63], it also regulates aggression in some species [61, 64, 65]. In honeybees and fruit flies, DA also regulates the locomotor activity [66–68], which diverges among termite castes. Considering these findings, precise comparison of 5-HT and DA systems among termite castes may be also fruitful.

The evolution of animal sociality is considered to occur by co-options of molecular and physiological mechanisms that already exist in ancestral solitary animals [69]. Eusocial termites are thought to have evolved from a subsocial (parent-offspring grouping) ancestor, like modern wood roaches *Cryptocercus* [70]. Given that basic physiological mechanisms like aminergic systems are common to both solitary and social insects, comparison of TA/OA and the other aminergic systems between termites and subsocial wood roaches or solitary cockroaches may elucidate the neurophysiological mechanisms leading to the evolution of eusociality in the termite lineage.

## Supporting Information

S1 Fig Examples of the original trace for the HPLC measurements. HPLC traces of a soldier and a pseudergates are shown by black and grey lines. The peaks of a chemical are identified as octopamine (OA), 3, 4-dihydroxybenzylamine (DHBA), dopamine (DA), B-acetyltyramine (NATA), tyramine (TA), tryptophan (Trp), and 5-hydroxytryptamine (5-HT).
(EPS)

S2 Fig The standardized levels of biogenic amines in soldiers and pseudergates. Tyramine (A), octopamine (B), dopamine (C) and 5-HT (D) levels in pseudergates (light grey) and soldiers (dark grey) (N = 23 each). The circles indicate the aminergic levels in each termite individual. Box plots show median values (solid horizontal line), 50th percentile values (box outline), 90th percentile values (whiskers), and outlier values (crosses). Asterisks indicate statistical significance (***p < 0.001, **p < 0.01 in exact Wilcoxon rank sum test).
(EPS)

S1 Movie Projection pattern of TA-l-ir neurons in pseudergates. Immunostaining using an anti-tyramine antibody identified TA-l-ir neurons and their fibers.
(AVI)

S1 Table Sizes of brain and SOG in *Hodotermopmsis sjostedti* [29]. (DOC)
