# Supplementary material for: Tyraminergic and Octopaminergic Modulation of Defensive Behavior in Termite Soldier
Source: PLoS One. 2016 May 19;11(5):e0154230. doi: 10.1371/journal.pone.0154230 (PMC4873212; doi:10.1371/journal.pone.0154230)
Supplement: S1 Table — (DOC) [file pone.0154230.s004.doc]

**Supplementary information**

**S1 Table. Sizes of brain and SOG in *Hodotermopmsis sjostedti* [29]*.***

| Individual | Caste | Brain width (BW) | Brain length (BL) | SOG width (SW) | SOG length (SL) | Brain-size index | SOG-size index |
| --- | --- | --- | --- | --- | --- | --- | --- |
| 1 | Soldier | 1.10 | 0.75 | 0.56 | 0.76 | 0.75 | 0.12 |
| 2 | Soldier | 1.05 | 0.68 | 0.51 | 0.67 | 0.61 | 0.09 |
| 3 | Soldier | 1.10 | 0.67 | 0.48 | 0.71 | 0.62 | 0.09 |
| 4 | Soldier | 1.08 | 0.69 | 0.49 | 0.73 | 0.65 | 0.09 |
| 5 | Soldier | 1.04 | 0.67 | 0.51 | 0.73 | 0.58 | 0.10 |
| 6 | Soldier | 1.02 | 0.60 | 0.52 | 0.69 | 0.49 | 0.10 |
| 7 | Soldier | 1.08 | 0.72 | 0.51 | 0.69 | 0.68 | 0.09 |
| 8 | Soldier | 1.02 | 0.69 | 0.50 | 0.66 | 0.60 | 0.09 |
| 9 | Soldier | 1.02 | 0.65 | 0.51 | 0.74 | 0.54 | 0.10 |
| 10 | Soldier | 1.03 | 0.65 | 0.50 | 0.70 | 0.54 | 0.09 |
| 1 | Pseudergate | 1.03 | 0.69 | 0.47 | 0.59 | 0.60 | 0.07 |
| 2 | Pseudergate | 1.07 | 0.70 | 0.45 | 0.60 | 0.65 | 0.06 |
| 3 | Pseudergate | 1.01 | 0.70 | 0.42 | 0.56 | 0.60 | 0.05 |
| 4 | Pseudergate | 1.04 | 0.70 | 0.46 | 0.55 | 0.61 | 0.06 |
| 5 | Pseudergate | 1.09 | 0.70 | 0.47 | 0.59 | 0.66 | 0.07 |
| 6 | Pseudergate | 1.03 | 0.72 | 0.43 | 0.59 | 0.63 | 0.06 |
| 7 | Pseudergate | 1.07 | 0.73 | 0.45 | 0.61 | 0.69 | 0.07 |
| 8 | Pseudergate | 1.07 | 0.68 | 0.46 | 0.59 | 0.62 | 0.06 |
| 9 | Pseudergate | 1.01 | 0.66 | 0.48 | 0.60 | 0.54 | 0.07 |
| 10 | Pseudergate | 1.01 | 0.68 | 0.47 | 0.62 | 0.57 | 0.07 |
